# Blocking ERK1/2 signaling impairs TGF-β1 tumor promoting function but enhances its tumor suppressing role in intrahepatic cholangiocarcinoma cells

**DOI:** 10.1186/s12935-017-0454-2

**Published:** 2017-09-26

**Authors:** Phaijit Sritananuwat, Natthaporn Sueangoen, Parichut Thummarati, Kittiya Islam, Tuangporn Suthiphongchai

**Affiliations:** 1 Department of Biochemistry, Faculty of Science, Mahidol University, Bangkok, 10400 Thailand; 2 Present Address: Faculty of Pharmaceutical Sciences, Ubon Ratchathani University, Ubon Ratchathani, Thailand; 3 Present Address: Research Center, Faculty of Medicine Ramathibodi Hospital, Mahidol University, Bangkok, Thailand

**Keywords:** Cholangiocarcinoma, ERK1/2, Invasion, Slug, Smad2/3, TGF-β1

## Abstract

**Background:**

Transforming growth factor-β (TGF-β) plays a paradoxical role in cancer: it suppresses proliferation at early stages but promotes metastasis at late stages. This cytokine is upregulated in cholangiocarcinoma and is implicated in cholangiocarcinoma invasion and metastasis. Here we investigated the roles of non-Smad pathway (ERK1/2) and Smad in TGF-β tumor promoting and suppressing activities in intrahepatic cholangiocarcinoma (ICC) cells.

**Methods:**

TGF-β1 effects on proliferation, invasion and migration of ICC cells, KKU-M213 and/or HuCCA-1, were investigated using MTT, colony formation, in vitro Transwell and wound healing assays. Levels of mRNAs and proteins/phospho-proteins were measured by quantitative (q)RT-PCR and Western blotting respectively. E-cadherin localization was examined by immunofluorescence and secreted MMP-9 activity was assayed by gelatin zymography. The role of ERK1/2 signaling was evaluated by treating cells with TGF-β1 in combination with MEK1/2 inhibitor U0126, and that of Smad2/3 and Slug using siSmad2/3- and siSlug-transfected cells.

**Results:**

h-TGF-β1 enhanced KKU-M213 cell invasion and migration and induced epithelial-mesenchymal transition as shown by an increase in vimentin, Slug and secreted MMP-9 levels and by a change in E-cadherin localization from membrane to cytosol, while retaining the cytokine’s ability to attenuate cell proliferation. h-TGF-β1 stimulated Smad2/3 and ERK1/2 phosphorylation, and the MEK1/2 inhibitor U0126 attenuated TGF-β1-induced KKU-M213 cell invasion and MMP-9 production but moderately enhanced the cytokine growth inhibitory activity. The latter effect was more noticeable in HuCCA-1 cells, which resisted TGF-β-anti-proliferative activity. Smad2/3 knock-down suppressed TGF-β1 ability to induce ERK1/2 phosphorylation, Slug expression and cell invasion, whereas Slug knock-down suppressed cell invasion and vimentin expression but marginally affected ERK1/2 activation and MMP-9 secretion. These results indicate that TGF-β1 activated ERK1/2 through Smad2/3 but not Slug pathway, and that ERK1/2 enhanced TGF-β1 tumor promoting but repressed its tumor suppressing functions.

**Conclusions:**

Inhibiting ERK1/2 activation attenuates TGF-β1 tumor promoting effect (invasion) but retains its tumor suppressing role, thereby highlighting the importance of ERK1/2 in resolving the TGF-β paradox switch.

**Electronic supplementary material:**

The online version of this article (doi:10.1186/s12935-017-0454-2) contains supplementary material, which is available to authorized users.

## Background

Cholangiocarcinoma (CCA), a bile duct malignancy, is one of the most severe forms of cancer with a 5-year survival rate < 5% [1]. Although intrahepatic CCA (ICC) is not frequently found worldwide, its incidence has increased during the past decades [2]. Incidence of ICC is high in Asia [3], particularly in northeast Thailand (96 per 100,000 in males, about 100 times higher than that in USA and Europe) [4].

Risk factors of ICC include hepatolithiasis, primary sclerosis cholangitis, cirrhosis, and liver fluke (*Opisthorchis viverrini* or *Clonorchis sinensis*) infection, depending on geographical location [4]. Interestingly, the majority of these risk factors are those associated with inflammation. In Southeast Asia, including northeast Thailand, *O*. *viverrini* infection is the major risk factor for CCA [5]. In a hamster model, this parasite damages bile duct epithelia, initiates inflammation, enhances peribiliary fibrosis, and increases transforming growth factor (TGF)-β, IL-1β and TNF-α levels [6, 7]. In addition, exposure of *O*. *viverrini*-infected hamsters to N-nitrosodimethylamine promotes liver fibrosis and cholangiocarcinogenesis [8].

TGF-β regulates a diversity of cellular functions, such as embryogenesis, cell proliferation, inflammation, and fibrogenesis [9]. During liver injury, TGF-β expression is up-regulated and promotes liver fibrosis [10]. However in carcinogenesis, TGF-β initially functions as a tumor suppressor in the early stages of the disease, but acts as a tumor promoter at the later stages [11]. This change in TGF-β from being a tumor suppressor to a tumor promoter is known as ‘TGF-β paradox’ switch.

In the early stages of cancer development, TGF-β inhibition of cell proliferation and induction of apoptosis occur via a variety of mechanisms, viz., enhancing levels of pro-apoptotic Bcl (Bim) or cyclin-dependent kinase inhibitors, or suppressing those of Myc, Id1/2 or survivin [12, 13]. However, during cancer development, cells frequently lose their response to TGF-β growth inhibitory activity, either through mutations in TGF-β signaling mediators, such as TGF-β receptor type II (TβRII), Smad2 and Smad4 [13], or through alterations in cell cycle regulatory proteins, viz., down-regulation of p53, p21 and pRb [14] or up-regulation of Myc, Akt, Ras/ERK1/2 (extracellular signal regulated kinase 1/2) [13]. At these later stages, cancer cells become responsive to the TGF-β tumor promoting activity that enhances tumor invasiveness and metastasis via an epithelial-mesenchymal transition (EMT) process [15]. In general, TGF-β conveys signals through phosphorylation of Smad2/3, but it also can transduce signals via Ras/ERK1/2, PI3 K, p38, and RhoA pathways, the latter processes augmenting its tumor promoting activity [16–19].

TGF-β1 is overexpressed in biliary dysplasia, hyperplasia and ICC [20] and is implicated in both initiation and progression of cholangiocarcinogenesis [21, 22]. Overexpression of cyclin D [20] and loss of Smad4 [21] contribute to ICC ability to escape from TGF-β growth inhibitory activity during the initial stages of tumorigenesis [20]. Besides, TGF-β enhances cell invasiveness and EMT in CCA cell lines [22] and its level in ICC patients correlates with clinical stages and metastasis to lymph nodes [23]. These findings indicate the existence of a ‘TGF-β paradox’ switch in CCA.

ERK and its upstream regulators, namely, Ras, B-Raf and growth factor receptors, frequently are mutated and participate in cancer progression [24]. Upregulation of Ras/ERK1/2 pathway has commonly reported in ICC as well [25]. Although expression of ERK and TGF-β have been observed in CCA, the interaction of these pathways and the role of ERK pathway in ICC under TGF-β enriched environment remain unclear. We posit the notion that in TGF-β-responsive ICC, ERK pathway plays a pivotal role in resolving the ‘TGF-β paradox’. Towards this goal, we demonstrate that in an ICC cell line, TGF-β induces ERK phosphorylation via Smad pathway and blocking this activation with U0126, a MEK1/2 inhibitor, results in the inhibition of TGF-β tumor promoting activity and the enhancement of its anti-proliferative role.

## Materials and methods

### Cell culture

Human *O*. *viverrini*-associated ICC cell lines, HuCCA-1 and KKU-M213, derived from cells of Thai CCA patients, were generously provided by Professor Stitaya Sirisinha (Mahidol University, Bangkok, Thailand) and Professor Banchob Sripa (Khon Kaen University, Khon Kaen, Thailand) respectively. Cells were maintained at 37 °C in HAM’s F-12 media (GIBCO, Grand Island, NY) consisting of 15 mM HEPES, 14 mM NaHCO_3_, 100 U/mL penicillin G, 100 μg/mL streptomycin, and 10% heat-inactivated fetal bovine serum (FBS) under a humidified 5% CO_2_ atmosphere.

### MTT and clonogenic cell proliferation assays

For MTT assay, ICC cells (3 × 10^3^) were plated onto a 96-well plate in 10% FBS media and cultured for 24 h, then treated with 5 ng/mL h-TGF-β1 (R&D Systems, Minneapolis, MN), or 10 μM SB431542 (TβRI kinase inhibitor) (Cayman, Ann Arbor, MI), or 1 μM U0126 (MEK1/2 inhibitor) (Tocris, Bristol, UK), or a combination of h-TGF-β1 and each inhibitor in 10% FBS media for up to 72 h. Control cells were treated with vehicle (4 µM HCl, 0.001% bovine serum albumin (BSA) and 0.025% dimethyl sulfoxide (DMSO)). At indicated times, the numbers of viable cells were determined by replacing media with 100 μL of 0.5 mg/mL 3-(4,5-dimethylthiazol-2-yl)-2,5-diphenyltetrazolium bromide (AppliChem, Darmstadt, Germany) in 10% FBS media. Cells were incubated for 4 h, then supernatant was removed and insoluble formazan dye was dissolved in 200 µL of DMSO. A 540 nm was measured using a Multiskan EX Microplate Reader (Thermo Labsystems, Finland).

In the clonogenic assay, 200 ICC cells were plated onto a 24-well plate in 10% FBS media, then treated with TGF-β in the presence and absence of inhibitor. After 7 days, cells were fixed with 4% paraformaldehyde and stained with 0.5% (w/v) crystal violet in 25% methanol. Colony forming ability was determined by measuring the area of crystal violet stain using an Image J program (NIH, Bethesda, MD).

### *In vitro* wound healing assay

Confluent cells in a 24-well plate were incubated for 24 h with 0.1% FBS media containing h-TGF-β1 with and without SB431542. Control cells were treated with vehicle. Wounds were created by scratching with a 1-mL-pipet tip and wells were washed with 0.1% FBS media, then 0.1% FBS media containing h-TGF-β1 with and without inhibitor were added. Cells were incubated for 24 h to allow migration into wound area. Images under phase contrast microscope (40 × magnification) were recorded and analyzed using an Image J program at 0 h (immediately after wound creation) and at 24 h.

### Transwell in vitro invasion and migration assays

In vitro invasion and migration abilities of ICC cells were determined using Matrigel-coated and -uncoated Transwells (Corning Inc., Corning, NY) respectively. In the in vitro invasion assay, the upper surface of a Transwell polycarbonate membrane (6.5-mm diameter) was coated with 30 μg of Matrigel (BD Biosciences, Bedford, MA). Then a 200 µL aliquot of 10^5^ ICC cells (pre-incubated with h-TGF-β1, SB431542, U0126, or a combination of h-TGF-β1 with each inhibitor in 0.1% FBS media for 24 h, trypsinized and re-suspended in the same media) was added to the upper chamber, while the lower chamber was filled with the same media. Following incubation at 37 °C for 6 or 12 h, non-invaded cells in the upper chamber were removed and invaded cells attached underneath the membrane were stained with 5% crystal violet in 25% methanol. The numbers of invaded cells were counted in five random fields under a light microscope (100 × magnification).

### Quantitative (q)RT-PCR assay

ICC cells (80% confluent) were treated with h-TGF-β1 in 0.1% FBS media with or without SB431542 for 6 and 24 h. RNA was extracted using Illustra RNAspin Mini RNA Isolation Kit (GE Healthcare, Munchen, Germany). Total RNA (1 µg) was reverse transcribed using a random hexamer primer and Improm-II™ reverse transcriptase (Promega BioSciences, Madison, WI). QRT-PCR mixture (20-µL) reaction contained 1 × FastStart Universal SYBR Green Master cocktail (Roche Diagnostics, Mannheim, Germany) and 6 pmol of each specific primer pair (5′-CAGGTGGACCAGCTAACCAA-3′/5′-TGCCAGAGACGCATTGTCA-3′, 5′-CACGACGTCTTCCAGTACCGAGA-3′/5′-CATAGGTCACGTAGCCCACTTGGT-3′, and 5′-GTAACCCGTTGAACCCCATT-3′/5′-CCATCCAATCGGTAGTAGCG-3′ for vimentin and MMP-9 mRNA and 18S rRNA (internal control), respectively). Thermocycling was performed in Stratagene Mx 3000P instrument (Agilent Technologies, Santa Clara, CA) as follows: 95 °C for 10 min; and 40 cycles of 94 °C for 30 s, 58 °C for 1 min and 72 °C for 1 min. Relative mRNA levels of treated compared to untreated control cells were determined using 2^−ΔΔCt^ method [26].

Steady-state mRNA levels of TGF-β, activin and nodal were quantified by qRT-PCR as described above using RNA extracted from 80% confluent cells cultured in 10% FBS media. Specific primer pair for TGF-β, activin and nodal was 5′-AACCCACCCGAAATCTATGAC-3′/5′-GCTGAGGTATCGCCAGGAAT-3′, 5′-GGAGCTCAGACAGCTCTTACC-3′/5′-GCAAATTCTCTTTCTGGTCCCC-3′, and 5′-AGACATCATCCGCAGCCTACA-3′/5′-GTCCATCTGAAACCGCTCTAAG-3′, respectively [27, 28].

### Western blotting assay

ICC cells (80% confluent) were treated with h-TGF-β1 with or without SB431542 or U0126 in 0.1% FBS media. At indicated times, cells were lysed with a solution of 150 mM Tris–HCl pH 7.4, containing 150 mM NaCl, 5 mM EGTA, 5 mM EDTA, 0.1% SDS 1% sodium deoxycholate, 1% Nonidet P-40, 1X protease inhibitor cocktail (Roche Diagnostics), 50 mM NaF, 2 mM Na_3_VO_4_, 40 mM β-glycerophosphate, and 1 mM dithiothreitol, then subjected to 10% SDS-PAGE. Proteins were transferred onto nitrocellulose membrane and incubated with primary antibodies against phospho-Smad2, phospho-ERK1/2, total-ERK1/2 (Cell Signaling Technology, Danvers, MA), vimentin, Slug, β-actin and GAPDH (loading control) (Santa Cruz Biotechnology, Santa Cruz, CA), followed by horse-radish peroxidase-conjugated anti-IgG secondary antibodies (Santa Cruz Biotechnology). Chemiluminescent signals of immunoreactive proteins were visualized using Luminata™ Forte Western HRP substrate (Millipore, Billerica, MA) and recorded by a G-Box Chemi XL system (Syngene, Cambridge, UK).

### Gelatinase and urokinase plasminogen activator (uPA) zymography

Amounts of MMP-2 and MMP-9 in conditioned media were determined by gelatin zymography and uPA amount by plasminogen-gelatin zymography. Cells (10^5^) were treated with h-TGF-β1 with and without SB431542 or U0126 in 0.1% FBS media. At indicated times, 20 μL aliquots of conditioned media were mixed with non-reducing SDS-PAGE loading buffer and subjected to 7.5% SDS-PAGE containing 1 mg/mL gelatin for detection of MMP-2 and -9, or 10% SDS-PAGE containing 1 mg/mL gelatin plus 10 μg/mL plasminogen for uPA detection. After washing with 2.5% Triton X-100, gels were incubated for 18 h in a solution containing 50 mM Tris–HCl pH 7.5, 10 mM CaCl_2_, 1 μM ZnCl_2_ and 1% Triton X-100 for gelatinase assay or 100 mM Tris–HCl pH 7.8, 150 mM NaCl and 1% Triton X-100 for uPA assay. Gels were stained with Coomassie blue dye and clear band of 67 and 82kDa in gelatin gel corresponds to MMP-2 and -9 respectively, and of 43 kDa in plasminogen-gelatin gel to that of uPA.

### Immunofluorescence cell staining

ICC cells (60% confluent) were deposited on coverslips and treated for 24 h with h-TGF-β1 with and without SB431542 or U0126 in 0.1% FBS media, then incubated with phosphate-buffered saline (PBS) containing 4% paraformaldehyde and 2% sucrose, and permeabilized with 0.25% Triton X-100 followed by incubation with 2% BSA in PBS. Cells then were treated with mouse anti-E-cadherin antibody (Santa Cruz Biotechnology), followed by Alexa Fluor^®^ 488-conjugated anti-mouse IgG secondary antibody (Molecular Probes^®^, Eugene, OR). Cell nuclei were stained with DAPI (Molecular Probes^®^). Coverslips were mounted with Prolong^®^ gold antifade reagent (Molecular Probes^®^) and examined under a confocal microscope (Olympus FV10; Olympus Corp., Tokyo, Japan).

### Small interfering (si)RNA cell transfection

ICC cells (2.5 × 10^5^) in 10% FBS media were cultured in a 6-well plate for 24 h, then transfected with 15 nM siRNA targeting mRNA of MMP-9 (siMMP-9), Smad2/3 (siSmad2/3), Slug (siSlug) (Santa Cruz Biotechnology) or 15 nM AllStars siRNA Negative Control (siNeg) (Qiagen, Valencia, CA) using Lipofectamin^®^ RNAiMAX reagent (Invitrogen, Carlsbad, CA) following manufacturer’s protocol. After 24 or 48 h, cells were treated with or without h-TGF-β1 in 0.1% FBS media for another 24 h. Invasive ability of treated cells then was determined using the Transwell assay as described above.

### Statistical analysis

Statistical analysis was performed using SPSS software version 16.0. Data are presented as mean ± standard error of mean (SEM) of three independent experiments conducted at least in duplicate. Statistical comparisons of more than two groups were performed using one-way analysis of variance (one-way ANOVA) with LSD’s post hoc test. Data are considered significantly different when *P* value < 0.05.

## Results

### Induction of migration and invasive abilities of ICC cells by h-TGF-β1

**Fig. 1:**
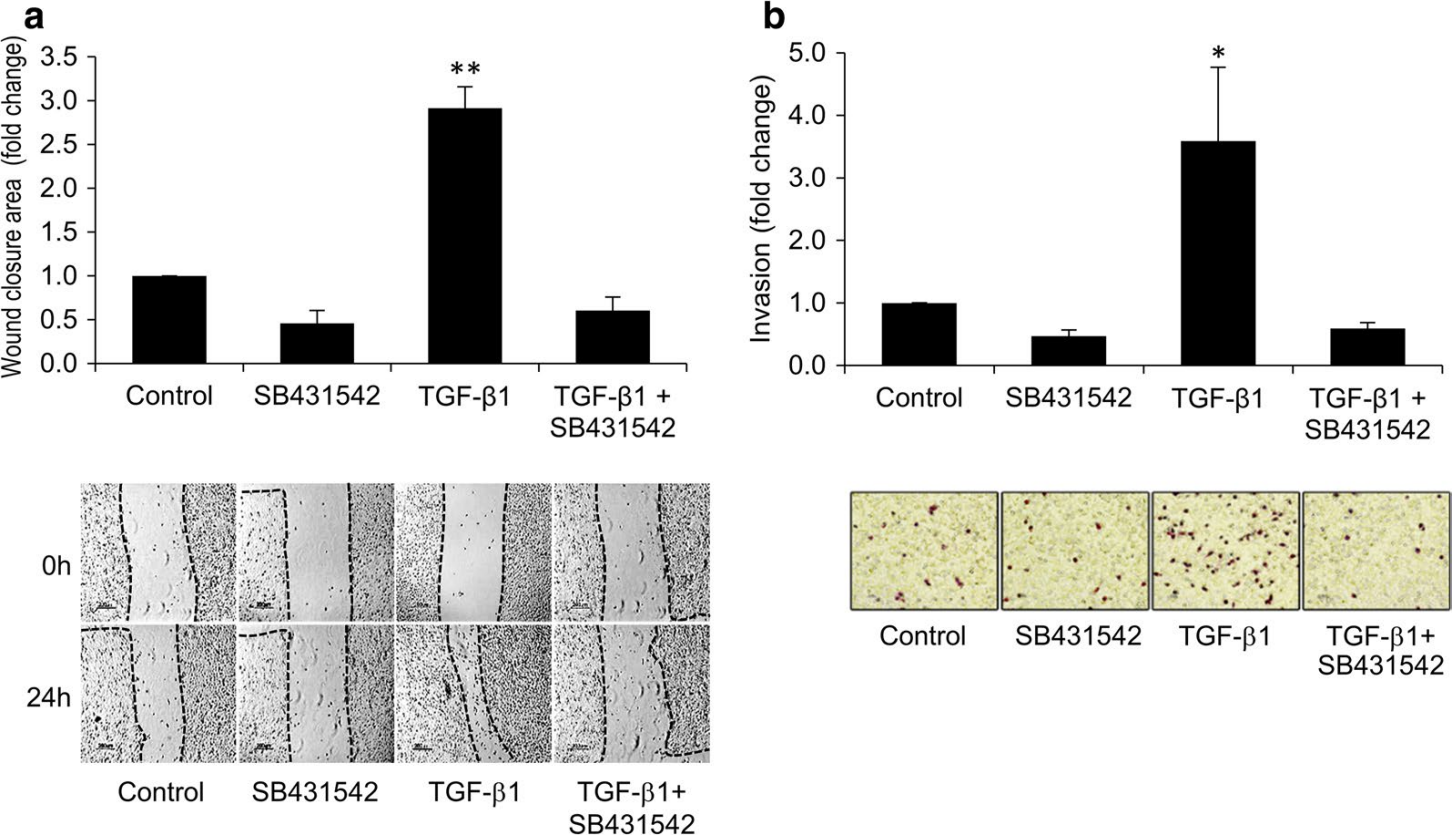
Effects of h-TGF-β1 on KKU-M213 cell migration and invasion. KKU-M213 cells were pre-treated with 5 ng/mL h-TGF-β1 with or without 10 µM SB431542 in 0.1% FBS media for 24 h before assaying for migration and invasion by wound healing and Transwell invasion technique respectively. In the wound healing assay, after creating the wound, cells were allowed to migrate for 24 h into the wound area using the same media as in pre-treatment condition (**a**). The area between the two dotted lines of each image (**a**) represents the wound area at 0 and 24 h. In the invasion assay, cells (10^5^) in the same media as those of pre-treated condition were plated onto the upper compartment of a Matrigel-coated Transwell and allowed to invade for 6 h, and invaded cells attached underneath of the membrane were stained with 5% crystal violet and counted (**b**). Graphs are presented as mean ± SEM of fold change in wound closure area quantitated by Image J program, or in numbers of invaded cells compared to controls (2100 ± 410 invaded cells/well) from at least three independent experiments. *P < 0.05, **P < 0.001

TGF-β1 is involved in invasion and metastasis of a number of cancers including CCA [22, 23]. This property was tested for an ICC cell line, KKU-M213, derived from tissue of a Thai ICC patient. Cell migration was evaluated using wound healing assay following treatment of KKU-M213 cells with 5 ng/mL h-TGF-β1 for 24 h. This resulted in a threefold increase in cell migration compared to untreated control, and this effect was abolished upon treatment with 10 µM TβRI inhibitor, SB431542 (Fig. 1a). Using a Transwell assay, h-TGF-β1 also significantly induced in vitro invasive ability of KKU-M213 cells, which also was inhibited by 10 µM SB431542 (Fig. 1b).

### Induction of ICC cell EMT and MMP-9 secretion by h-TGF-β1

**Fig. 2:**
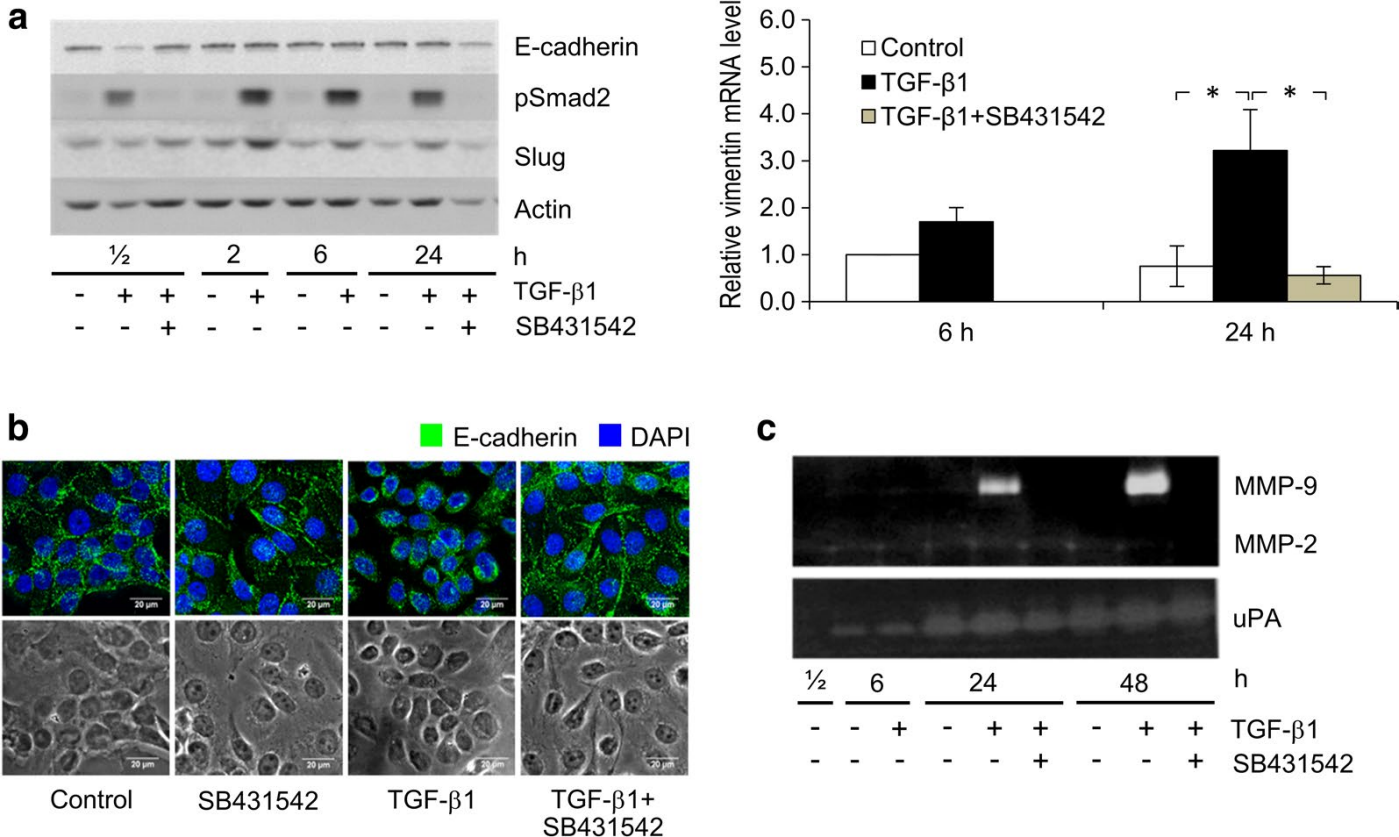
Induction by h-TGF-β1 of KKU-M213 EMT and MMP-9 secretion. KKU-M213 cells were treated with 5 ng/mL h-TGF-β1 with or without 10 µM SB431542 in 0.1% FBS media for the indicated times before assaying for EMT and MMP-9 expression. In EMT assay, cells were lysed and analyzed by immunoblotting using anti-E-cadherin, -phospho-Smad2, -Slug and -β-actin antibodies (**a**, left panel). The level of vimentin mRNA was determined by SYBR-based qRT-PCR and normalized relative to 18S rRNA (**a**, right panel). Relative mRNA levels were calculated using 2^−ΔΔCt^ formula compared to those of controls. After treatment for 24 h, E-cadherin localization was determined by immunofluorescence using anti-E-cadherin antibody (green) and DAPI (blue), then visualized under confocal microscopy (60 × objective lens with 2 × digital zoom), which shows that TGF-β changed E-cadherin localization from membrane to cytoplasm and SB431542 treatment reversed this effect (**b**). Phase contrast microscopy of the same field is shown in lower panel. MMP-2 and -9 in conditioned media from treated cells were analyzed by gelatin zymography and for uPA by plasminogen-gelatin zymography (**c**, upper panel). Results were obtained from three independent experiments. *﻿P ﻿< 0.05

TGF-β enhances tumor invasion and metastasis through promoting EMT, a process mediated by a group of transcription factors, such as Slug and Snail, via the canonical signaling Smad pathway [29, 30]. The existence of this TGF-β signaling pathway in KKU-M213 cells was demonstrated by Western blot detection of phospho-Smad2, which was induced within the first 30 min of treatment with 5 ng/mL h-TGF-β1 and was maintained for up to 24 h; phosphorylation was almost completely inhibited by 10 µM SB431542 (Fig. 2a).

**Fig. 3:**
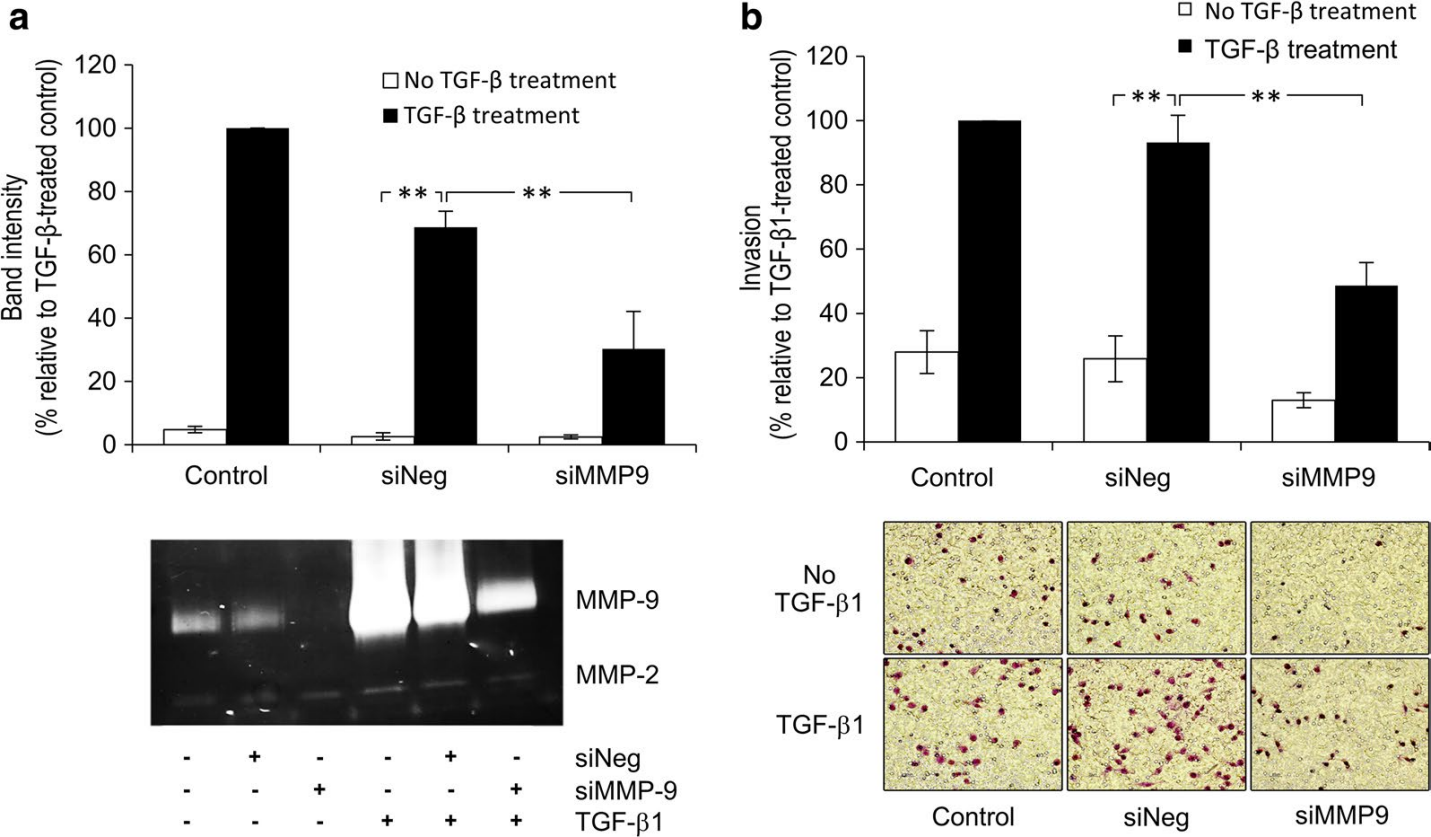
Effects of MMP-9 gene expression silencing on h-TGF-β1-induced KKU-M213 cell invasion. Cells were transfected with 15 nM siMMP-9 or non-targeting siRNA (siNeg) for 24 h followed by treatment with or without 5 ng/mL h-TGF-β1 for 24 h in 0.1% FBS media. Control experiment was performed using non-transfected cells. Silencing efficiency was evaluated from the reduction in band intensity of MMP-9 from gelatin zymogram (**a**). For in vitro invasion assay, cells (10^5^) were plated onto Matrigel-coated Transwell chamber and incubated for 6 h. Numbers of invaded cells were stained with 5% crystal violet and counted (**b**). Data are presented as mean ± SEM of percent band intensity (**a**) or percent invaded cells (**b**) relative to h-TGF-β1-treated control from at least three independent experiments. **P < 0.001

EMT induced by h-TGF-β1 was demonstrated by determining the increase in levels of Slug, E-cadherin, vimentin and matrix degrading enzymes. Slug level was raised 1.5–2.5 folds following 2–6 h of exposure to 5 ng/mL h-TGF-β1, which was maintained for up to 24 h (Fig. 2a). However, E-cadherin level remained unchanged during 24 h of h-TGF-β1 treatment, but was translocated from plasma membrane to cytosol (Fig. 2b). Vimentin mRNA level was induced 6 ± 2 folds by h-TGF-β1 (Fig. 2a). Zymography was employed to demonstrate that h-TGF-β1 elevated MMP-9 secretion into conditioned media, being apparent (sixfold increase) after 24 h and increased (14 folds) after another 24 h, but this phenomenon was nearly completely abolished by SB431542 (Fig. 2c). The cells also secreted small quantities of uPA and MMP-2, both of which were not affected significantly by exposure to h-TGF-β1 (Fig. 2c). Silencing MMP-9 gene expression by siMMP-9 reduced TGF-β1-induced MMP-9 secretion by approximately 60% (Fig. 3a). This silencing effect significantly inhibits both basal (by 50 ± 10%) and h-TGF-β1-activated invasiveness (by 48 ± 5%) (Fig. 3b).

### Involvement of ERK1/2 in h-TGF-β1-induced ICC cell invasiveness

**Fig. 4:**
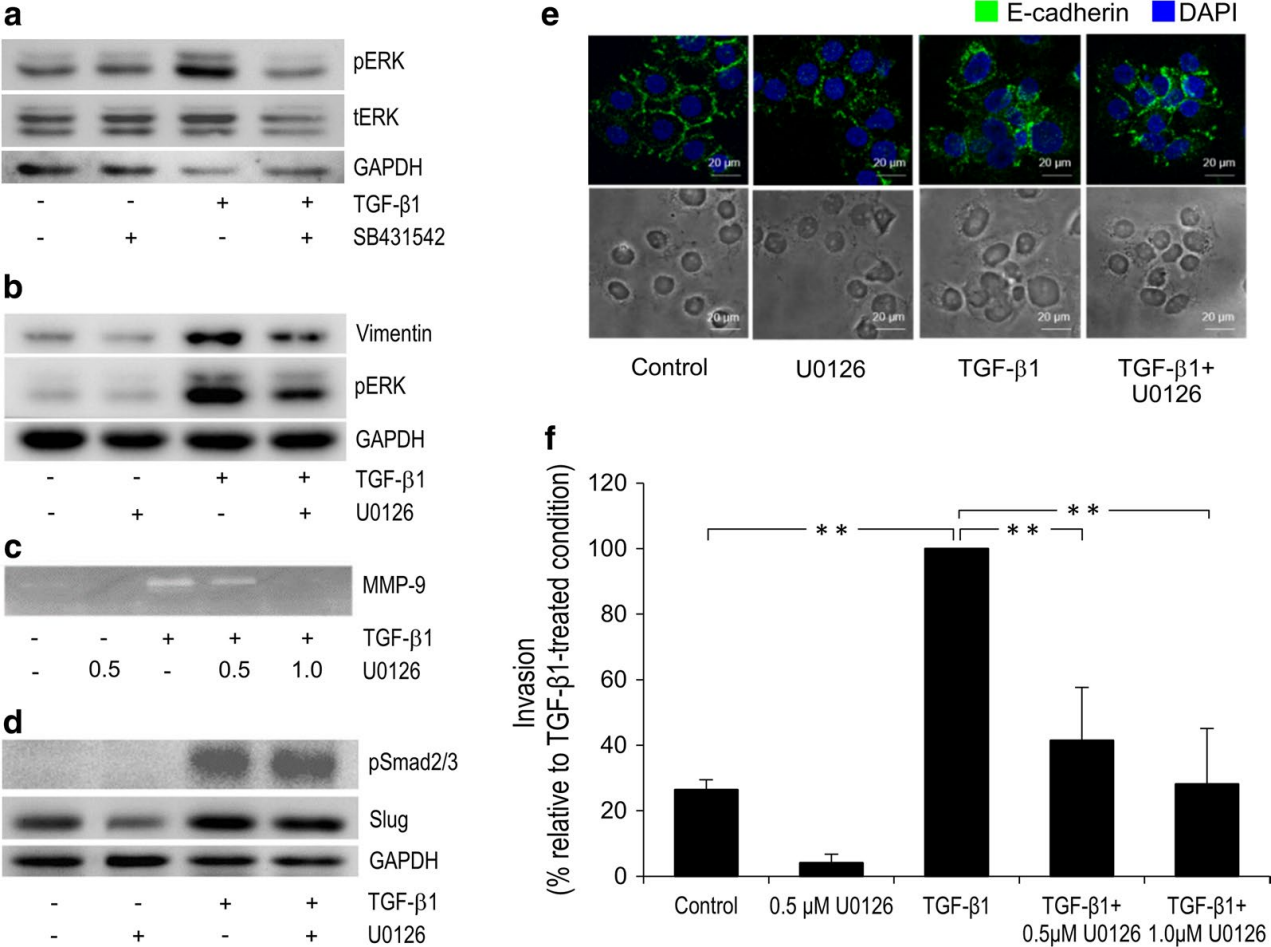
Effects of U0126 on h-TGF-β1-induced EMT and invasion of KKU-M213 cells. Cells were treated with 5 ng/mL h-TGF-β1 and/or 10 µM SB431542 or 1 µM U0126 in 0.1% FBS media for 24 h or for the indicated time before assay. Cell lysates were analyzed for levels of total ERK1/2 (tERK) (**a**), phospho-ERK1/2 (pERK) (**a**, **b**), vimentin (**b**) phospho-Smad and Slug (**d**) by immunoblotting (see Additional file 1: Figure 1 for quantitative data). Conditioned media were analyzed for MMP-9 by gelatin zymography (**c**). Immunoblot and zymography data are representative of three independent experiments. E-cadherin localization was examined by immunofluorescence staining with anti-E-cadherin antibody (green) and DAPI (blue), which show that U0126 reversed the effect of TGF-β induced reduction of E-cadherin membrane localization (**e**). For the invasion assay, cells (10^5^) in the same media as those of pre-treated condition were plated onto in vitro invasion Transwell chamber and allowed to invade for 6 h (**f**). Data are presented as mean ± SEM of percent change in number of invaded cells compared to h-TGF-β-treated condition obtained from three independent experiments. *P < 0.05, **P < 0.001

Not only Smad but ERK1/2 has been reported to act as downstream effectors contributing to TGF-β-induced EMT [31]. The notion that in ICC cells h-TGF-β1 also acts via MEK/ERK pathway was tested by investigating the effects of MEK1/2 inhibitor U0126 on h-TGF-β1-induced invasiveness of KKU-M213 cells. Exposure to 5 ng/mL h-TGF-β1 for 24 h resulted in 2- to 6-fold increase in ERK1/2 phosphorylation, which was inhibited by 10 µM SB431542 (Fig. 4a and Additional file 1: Figure 1a). After 24 h in the presence of 1 µM U0126, h-TGF-β1-induced vimentin expression was reduced 23 ± 9% (Fig. 4b and Additional file 1: Figure 1b) and MMP-9 secretion almost 75% (Fig. 4c), but not that of Smad2/3 phosphorylation or Slug expression (Fig. 4d). In addition, U0126 attenuated TGF-β ability to reduce E-cadherin membrane localization (Fig. 4e) and to induce invasion by approximately 70% (Fig. 4f). These results demonstrate that TGF-β1-induced KKU-M213 cell invasiveness through induction of EMT and MMP-9 secretion was, at least in part, through activation of ERK1/2 pathway. The effects of TGF-β and U0126 on invasion were also confirmed in another ICC cell line, HuCCA-1, which showed 2.5-fold increase in cell invasiveness upon TGF β stimulation. This effect could be inhibited (to some extent) by co-treatment with U0126 (Additional file 2: Figure 2). Interestingly in the absence of h-TGF-β1, invasiveness of both cell lines was also attenuated by U0126, indicating that ERK1/2 pathway was involved also in ICC cell basal invasiveness.

### Roles of Smad2/3 and slug in h-TGF-β1-induced ERK1/2 activation and invasiveness of ICC cells

**Fig. 5:**
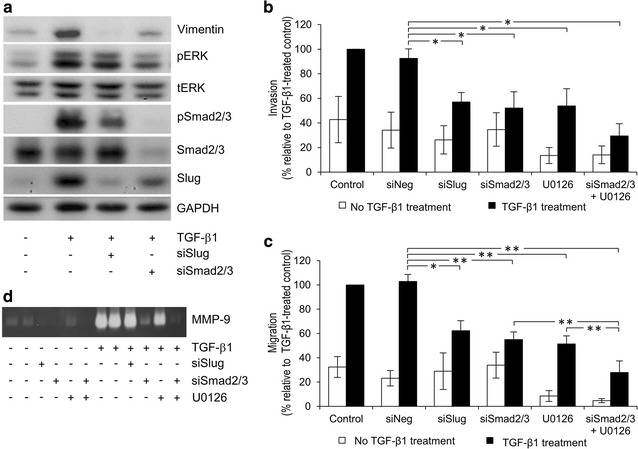
Effects of Smad2/3 and Slug silencing on ERK1/2 activation and biological processes in h-TGF-β1-induced KKU-M213 cells. Smad2/3 and Slug expression in KKU-M213 cells were inhibited using specific siRNA for 24 h. After 48 h of transfection, cells were treated with or without 5 ng/mL h-TGF-β1 in 0.1% FBS media for 24 h, followed by analysis for levels of specific proteins in the cell lysate by immunoblotting (**a**), cell migration (**b**) and invasion (**c**) by in vitro. Transwell assays and level of MMP-9 in conditioned media by gelatin zymography (**d**). For immunoblotting and zymography, data are representative of the results obtained from three independent experiments. Immunoblot quantitative data are shown in Additional file 3: Figure 3. For Transwell assay, data are presented as mean ± SEM of percent change in number of invaded cells compared to h-TGF-β-treated condition obtained from three independent experiments. *P < 0.05, **P < 0.001

Smad phosphorylation and Slug production are well-known downstream regulators of TGF-β1-induced progression of many types of cancers [29, 30]. Thus, we examined their roles in ERK1/2 activation and invasiveness of h-TGF-β1-induced KKU-M213 cell by knocking down Smad2/3 and Slug expression levels by means of siRNA. Knocking down Smad2/3 in KKU-M213 cells reduced h-TGF-β1 ability to induce ERK phosphorylation 35 ± 9%, but not in the case with Slug knock down cells (Fig. 5a and Additional file 3: Figure 3a). Smad2/3 silencing also suppressed Slug (30 ± 10%) and vimentin (50 ± 10%) expression levels (Fig. 5a and Additional file 3: Figure 3c and d), cell invasion (45 ± 8%) (Fig. 5b), cell migration (47 ± 4%) (Fig. 5c) and MMP-9 secretion (60 ± 15%) (Fig. 5d). Similarly, Slug silencing inhibited h-TGF-β1-induced KKU-M213 cell migration (40 ± 6%), cell invasion (39 ± 3%), vimentin expression (75 ± 15%) and Smad phosphorylation (26 ± 5%) (Fig. 5a–c and Additional file 3: Figure  3b and d) but only marginally affected MMP-9 secretion (Fig. 5d).

### Role of TGF- β1 in inhibition of ICC cell proliferation

**Fig. 6:**
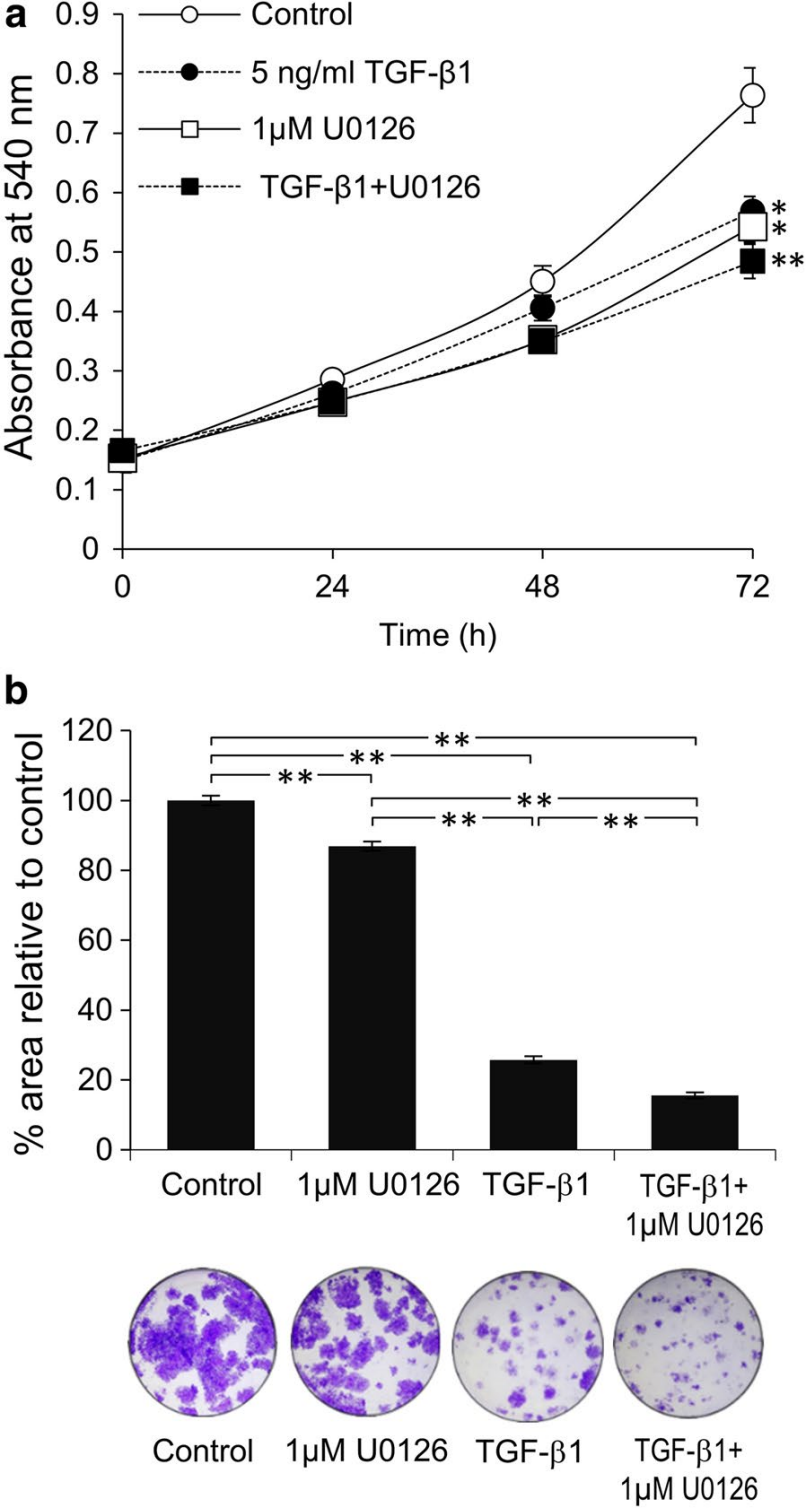
Role of ERK1/2 activation in h-TGF-β1 anti-proliferative activity of KKU-M213 cells. Cells were treated with 5 ng/mL h-TGF-β1 with or without 1 µM U0126 in 10% FBS media for indicated times and the numbers of viable cells were quantified using MTT assay (**a**). The graphs are presented as mean ± SEM from three independent experiments. *P < 0.05 and **P < 0.001 compared to control at 72 h. For the colony formation, 200 cells treated as those in MTT assay were incubated for 7 days and colony formation ability was quantified as percent ± SEM of crystal violet-stained area compared to control (**b**). ** *P* < 0.001

In general, TGF-β1 exhibits anti-proliferative and apoptotic activities during the early stages of tumorigenesis [12]. However, in the later stages of cancer progression, transformed cells are able to escape from this TGF-β1 growth inhibitory effect [13]. In order to determine if this latter phenomenon also applies in ICC, KKU-M213 cells were cultured in 10% FBS media and permitted to proliferate (4- to 5 folds) over a period of 72 h (Fig. 6a). Surprisingly, the proliferation is significantly reduced 25 ± 4% upon h-TGF-β1 (5 ng/mL) treatment (Fig. 6a). U0126 (1 µM) also possessed similar anti-proliferative effect and h-TGF-β1 in combination with inhibitor does not significantly enhance the effect of either one alone (Fig. 6a).

Colony formation assay was performed to investigate the long-term effect of h-TGF-β1 and U0126 on proliferation of the two ICC cell lines, KKU-M213 and HuCCA-1. The ability of cells to proliferate was quantitated from determination of colony area. KKU-M213 cells were sensitive to TGF-β anti-proliferative activity (74 ± 1% reduction in colony area after 7 days of TGF-β-treatment compared to control), while blocking ERK activation with U0126 alone only slightly reduced colony area (13 ± 1% compared to control), but interestingly, when combined the colony area was further diminished (84 ± 1% compared to control) (Fig. 6b). Similarly with HuCCA-1 cells, TGF-β produced 14 ± 5% reduction in colony area, U0126 treatment alone 36 ± 5% reduction and the combination of TGF-β and U0126 54 ± 2% reduction (Additional file 4: Figure 4). These results indicate that blocking ERK1/2 activation assisted in TGF-β anti-proliferative function in ICC cells.

## Discussion

TGF-β is involved in several steps of cancer progression from tumorigenesis to metastasis [11]. Although it acts as a tumor suppressor by inhibiting cell proliferation and inducing apoptosis at the early stages of carcinogenesis, TGF-β promotes cancer cell invasion and metastasis at the later stages [11, 32]. Therefore, an understanding of the signaling mechanism(s) by which these two contradictory effects of TGF-β might enable introduction of ways and means to inhibit specifically the undesirable cancer cell phenotype (invasion/metastasis) while maintaining the more desirable property (anti-proliferative).

ERK1/2 activation has been implicated in TGF-β-induced EMT and cell invasiveness. Inhibiting ERK1/2 signaling blocks TGF-β-induced EMT in normal murine mammary glands (NMuMG) cells [31] and NSCLC cells [33]. Overexpression of TGF-β1 and aberrant expressions of EMT-related proteins, namely E-cadherin, vimentin and Slug, in CCA patients are correlated with metastasis and short survival time [34–36]. In addition, mutations in MAPK/ERK pathway components, especially KRas, are relatively common in ICC and are associated with reduction in progression-free survival and overall survival of ICC patients [37]. Although TGF-β [22] and ERK1/2 [38] both participate in EMT and invasion in CCA, a link between these two signaling molecules remains unclear.

**Fig. 7:**
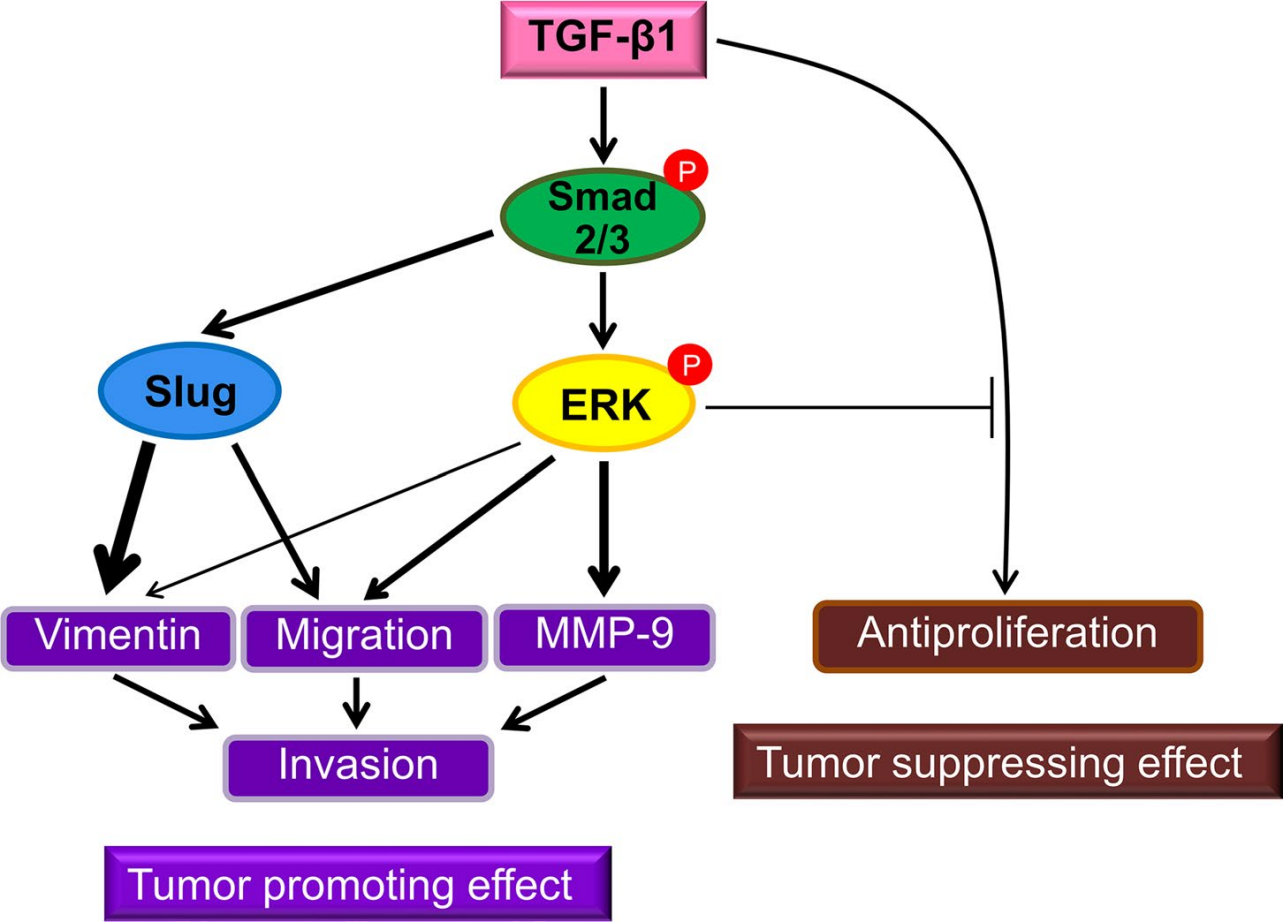
Diagram depicting roles of ERK1/2 activation in TGF-β1 tumor suppressor and tumor promoter effects in ICC cells

Using two human ICC cell lines, KKU-M213 and HuCCA-1, we demonstrate that h-TGF-β1 promotes cell invasion and that inhibition of ERK1/2 pathway suppresses this effect in both ICC cell lines with KKU-M213 being more sensitive to ERK1/2 inhibition. Focusing on the more responsive KKU-M213 cell line, h-TGF-β1 activates ERK1/2 signaling through the Smad2/3 but not Slug pathway. This ERK1/2 activation is partly responsible for the role of h-TGF-β1-induced ICC cell migration, EMT and MMP-9 expression. As regards TGF-β tumor suppression role, both ICC cell lines partially retain the cytokine anti-proliferative ability with KKU-M213 cells being more sensitive. Unlike in the case of cell invasion, blocking ERK pathway activation failed to attenuate TGF-β anti-proliferative activity and even exerted an additional effect on inhibiting cell proliferation. Taken together, these data indicate distinct roles of ERK1/2 in TGF-β1 tumor promoting and tumor suppressing effects in that ERK enhances TGF-β-role in promoting invasiveness but attenuates its growth inhibitory function in ICC cells (Fig. 7).

Ras/ERK pathway contributes to both TGF-β tumor suppressing (anti-proliferation and apoptosis) and tumor promoting (migration and invasion) activities [24]. For the latter, TGF-β induces EMT through upregulation of Ras/Raf/ERK1/2 pathway [31] and this in turn modulates expression of a set of genes involved in cell migration and ECM remodeling (viz., β4-integrins, laminins, RhoB, and MMPs) [39] and induces expression of c-fos, a component of AP-1 transcription factor, which promotes cell migration through activation of S100A2 [40]. In KKU-M213 cells, TGF-β1 activated both ERK and Smad2/3 pathways that act cooperatively to induce migration, invasion and MMP-9 secretion. This cooperativity might have resulted from crosstalk between the two pathways as AP-1, a mediator of ERK, can interact with Smad to form an EMT-promoting Smad complex (EPSC), which mediates expression of genes involved in the EMT process [41].

The effect of ERK signaling on TGF-β anti-proliferative activity depends on cell types. In p53-reconstituted lung cancer H1299 cells, ERK promotes anti-proliferation by inducing p21 expression through p53 (Ser 9) phosphorylation [42]. However, in Madin-Darby canine kidney cells prolonging hyperactivation of Raf, a positive regulator of ERK, down-regulates Smad3, causing resistance to TGF-β-induced growth arrest [43]. Here in ICC cells, ERK1/2 activation attenuated TGF-β anti-proliferative activity; however, the level of phospho-Smad was marginally affected by U0126 suggesting the role of Smad-independent pathways, such as AP-1 activation, to support cell proliferation in the presence of TGF-β. This notion is supported by the evidence in rat mesangial cells showing that TGF-β-activated ERK1/2 promotes expression of c-fos [44], which can enhance cell cycle progression through expressing cyclin D1 [45].

It is worth noting that the KKU-M213 cell line used in this study has basal migratory and invasive properties that are sensitive to SB431542, not only an inhibitor of TβRI (ALK-5) but also of ALK-4 and ALK-7, suggesting that the cells secrete and are responsive to TGF-β or its superfamily member(s), such as activin or nodal, which relay signals through their cognate receptors ALK-4 or ALK-7 [46]. This notion is supported by the qRT-PCR results showing expression of TGF-β and activin (but not nodal) in KKU-M213 cells (Additional file 5: Figure 5).

The mechanisms and kinetics of TGF-β-stimulated ERK1/2 activation are diverse depending on cell types and context. It may act through ShcA induction or Src phosphorylation, independent of Smad, leading to a rapid and transient (within minutes) activation of Ras/ERK pathway [47, 48]; or in an increase in expression of ERK pathway-dependent genes, such as EGFR, HB-EGF, PDGFR, and PDGF [49, 50]. This latter mechanism usually results in a delayed response (hours) and could be Smad-dependent [51]. In this study, ERK1/2 activation in KKU-M213 cell line required Smad2/3, but not Slug, and there was a delayed effect that could be retained for at least 24 h, implicating an indirect mechanism acting, in part, through the Smad pathway (Fig. 7).

## Conclusions

This study shows that in ICC cell lines, TGF-β1 stimulates cell migration and invasion (tumor promoting effects) and suppresses cell proliferation (tumor suppressing effect). The former function of TGF-β depends on ERK1/2 activation, whereas the anti-proliferative function is weakened by this pathway. This opens the door to target ERK-signaling pathway for selective suppression of ICC TGF-β-induced invasion and metastasis while enhancing the cytokine anti-proliferation function.

## Additional files

**Additional file 1.** Relative quantitative immunoblot of U0126 effect on total ERK, phospho-ERK1/2 and vimentin levels in h-TGF-β 1-induced ICC cells. Cells were treated with 5 ng/mL h-TGF- β 1 and/or 10 µM SB431542 or 1 µM U0126 in 0.1% FBS media for 24 h before analyzing for total ERK, phospho-ERK1/2 (a) and vimentin (b) by immunoblotting. Relative protein levels were analyzed from protein band intensity normalized relative to GAPDH and compared to those of untreated controls. Data are presented as mean ± SEM of fold change in protein levels relative to control. *P value < 0.05.

**Additional file 2.** Effects of U0126 on h-TGF-β1-induced HuCCA-1 cell invasion. Cells (10^5^) pre-treated with 5 ng/mL h-TGF-β1 and/or 1 µM U0126 in 0.1% FBS media for 24 h were plated onto in vitro invasion Transwell chamber and allowed to invade for 12 h. Invasion ability are presented as mean ± SEM of percent change in numbers of invaded cells compared to h-TGF-β-treated condition obtained from three independent experiments. *P value < 0.05, **P value < 0.001.

**Additional file 3.** Relative quantitative immunoblot of the effect of Smad and Slug silencing on ERK and Smad phosphorylation and Slug and vimentin expression in ICC cells. Smad2/3 and Slug expression in the ICC cells were suppressed using specific siRNA. After 48 h of transfection, cells were treated with or without 5 ng/mL h-TGF- β 1 in 0.1% FBS media for 24 h, followed by analysis for total and phospho-ERK1/2 (a), total and phospho-Smad2/3 (b), Slug (c) vimentin (d) and GAPDH levels by immunoblotting. Relative protein levels were analyzed from protein band intensity normalized relative to total ERK (a), total Smad 2/3 (b) or GAPDH (c, d) and compared to siNeg-transfected control. Data are presented as mean ± SEM of fold change in protein levels relative to control. *P value < 0.05, ^**^ *P* value < 0.001 compared to siNeg. ^*#*^ *P* value < 0.05, ^*##*^ *P* value < 0.001 compared to siNeg treated with TGF-β.

**Additional file 4.** Role of ERK1/2 activation in h-TGF-β1 anti-proliferative activity of HuCCA-1 cell line. Cells were treated with 5 ng/mL and 200 cells seeded on 24-well plate were treated with h-TGF-β1 with or without U0126 in 10% FBS media for 7 days. Colony formation ability was quantified as percent ± SEM of crystal violet-stained area compared to control. *P value < 0.05, ^**^ *P* value < 0.001.

**Additional file 5.** Steady state levels of TGF-β1, activin and nodal expression in KKU-M213 cells. Levels of TGF-β1, activin and nodal mRNA were determined by SYBR-green-based qRT-PCR using RNA extracted from 80% confluent cells cultured in 10% FBS media. Relative mRNA levels were calculated using 2^−ΔCt^ formula compared to that of 18 s rRNA.

## Abbreviations

TGF-β: transforming growth factor-β
ERK: extracellular signal-regulated kinase
ICC: intrahepatic cholangiocarcinoma
CCA: cholangiocarcinoma
TβRI: Type I TGFβ receptor
ALK: activin receptor-like kinase
GAPDH: glyceraldehyde 3-phosphate dehydrogenase
FBS: fetal bovine serum
mRNA: messenger RNA
qRT-PCR: quantitative reverse transcription polymerase chain reaction
MMP: matrix metalloproteinases
EMT: epithelial-mesenchymal transition
