# Supplementary figures and images for: Blocking ERK1/2 signaling impairs TGF-β1 tumor promoting function but enhances its tumor suppressing role in intrahepatic cholangiocarcinoma cells
Source: Cancer Cell Int. 2017 Sep 26;17:85. doi: 10.1186/s12935-017-0454-2 (PMC5615482; doi:10.1186/s12935-017-0454-2)

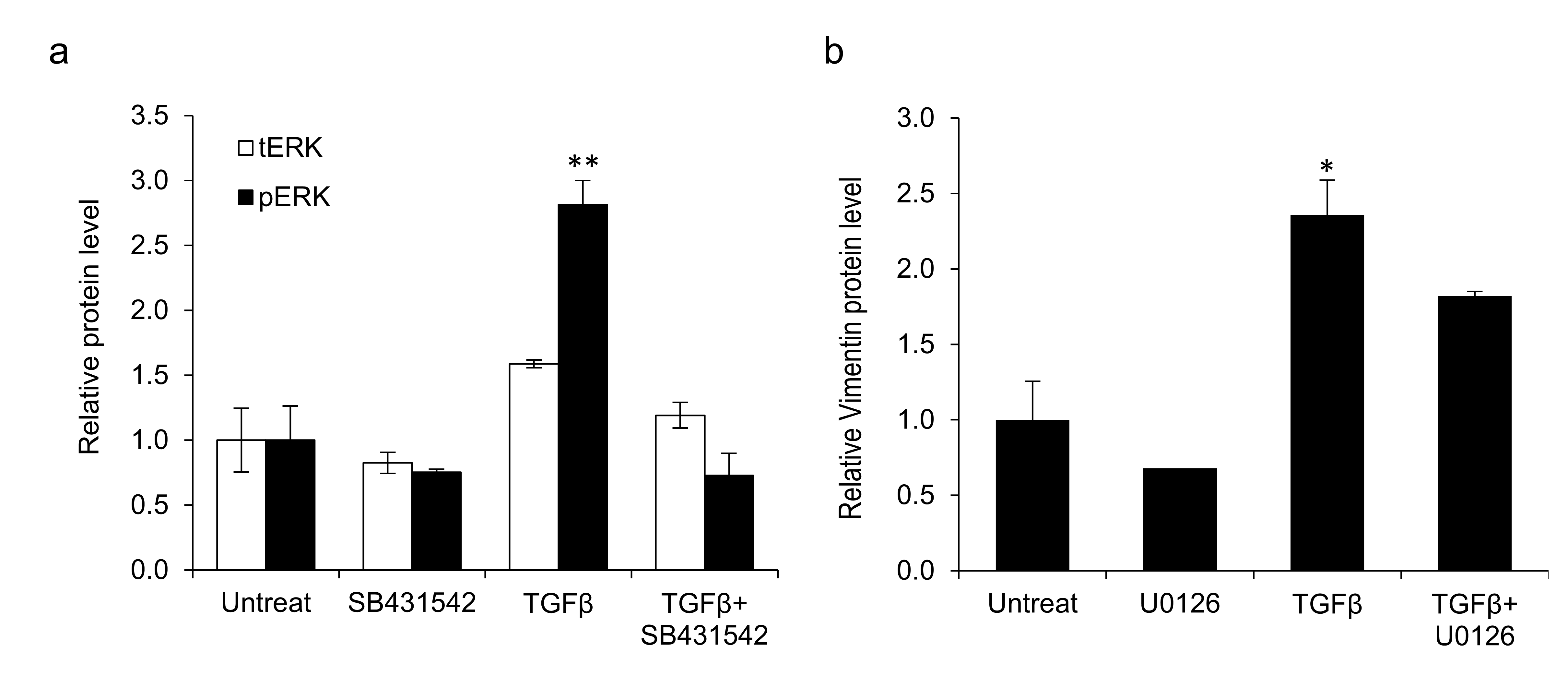

Supplement: Supplementary file 1 — Additional file 1. Relative quantitative immunoblot of U0126 effect on total ERK, phospho-ERK1/2 and vimentin levels in h-TGF-β 1-induced ICC cells. Cells were treated with 5 ng/mL h-TGF- β 1 and/or 10 µM SB431542 or 1 µM U0126 in 0.1% FBS media for 24 h before analyzing for total ERK, phospho-ERK1/2 (a) and vimentin (b) by immunoblotting. Relative protein levels were analyzed from protein band intensity normalized relative to GAPDH and compared to those of untreated controls. Data are presented as mean ± SEM of fold change in protein levels relative to control. *P value < 0.05. [file 12935_2017_454_MOESM1_ESM.tif]

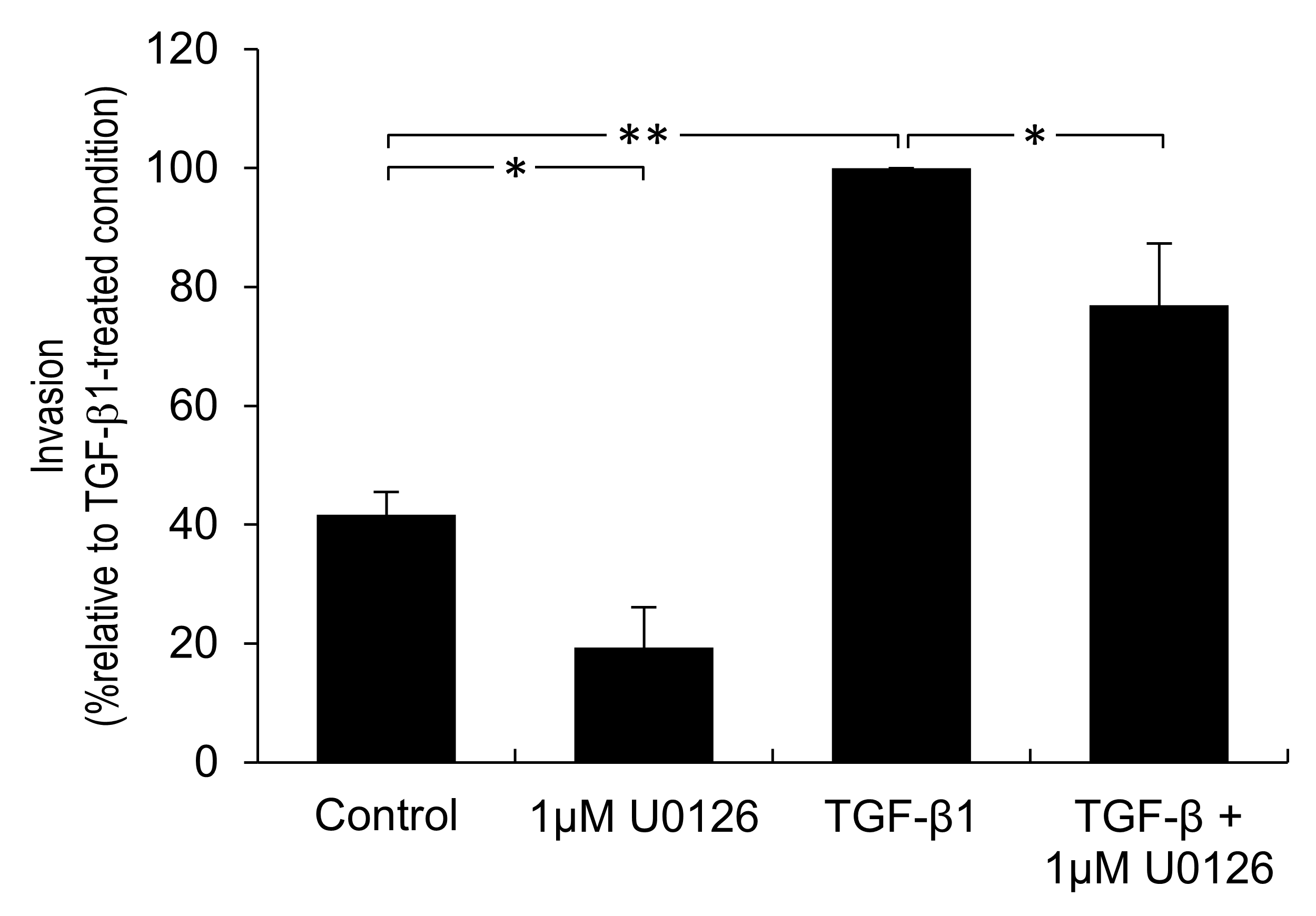

Supplement: Supplementary file 2 — Additional file 2. Effects of U0126 on h-TGF-β1-induced HuCCA-1 cell invasion. Cells (105) pre-treated with 5 ng/mL h-TGF-β1 and/or 1 µM U0126 in 0.1% FBS media for 24 h were plated onto in vitro invasion Transwell chamber and allowed to invade for 12 h. Invasion ability are presented as mean ± SEM of percent change in numbers of invaded cells compared to h-TGF-β-treated condition obtained from three independent experiments. *P value < 0.05, **P value < 0.001. [file 12935_2017_454_MOESM2_ESM.tif]

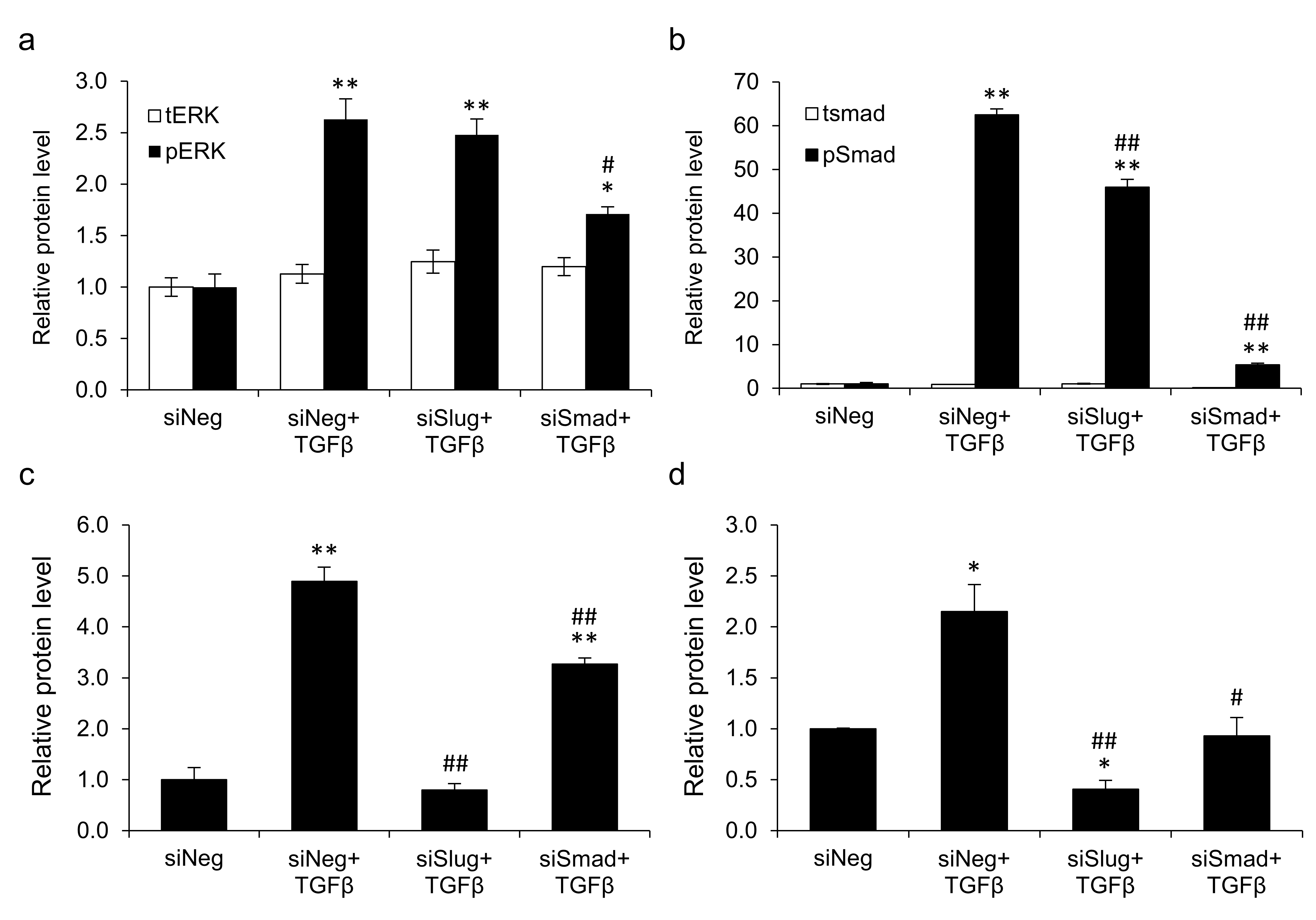

Supplement: Supplementary file 3 — Additional file 3. Relative quantitative immunoblot of the effect of Smad and Slug silencing on ERK and Smad phosphorylation and Slug and vimentin expression in ICC cells. Smad2/3 and Slug expression in the ICC cells were suppressed using specific siRNA. After 48 h of transfection, cells were treated with or without 5 ng/mL h-TGF- β 1 in 0.1% FBS media for 24 h, followed by analysis for total and phospho-ERK1/2 (a), total and phospho-Smad2/3 (b), Slug (c) vimentin (d) and GAPDH levels by immunoblotting. Relative protein levels were analyzed from protein band intensity normalized relative to total ERK (a), total Smad 2/3 (b) or GAPDH (c, d) and compared to siNeg-transfected control. Data are presented as mean ± SEM of fold change in protein levels relative to control. *P value < 0.05, ** P value < 0.001 compared to siNeg. # P value < 0.05, ## P value < 0.001 compared to siNeg treated with TGF-β. [file 12935_2017_454_MOESM3_ESM.tif]

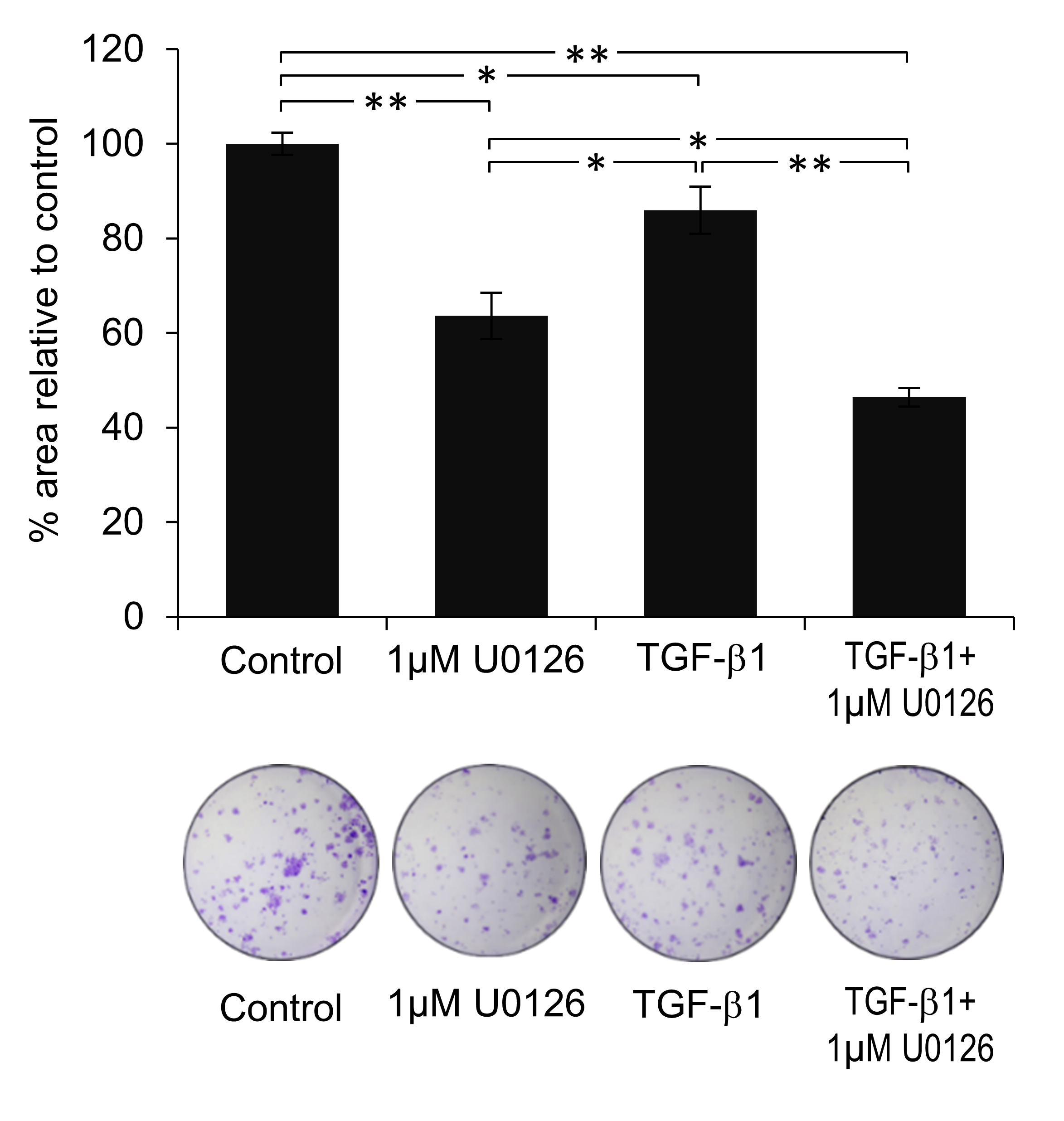

Supplement: Supplementary file 4 — Additional file 4. Role of ERK1/2 activation in h-TGF-β1 anti-proliferative activity of HuCCA-1 cell line. Cells were treated with 5 ng/mL and 200 cells seeded on 24-well plate were treated with h-TGF-β1 with or without U0126 in 10% FBS media for 7 days. Colony formation ability was quantified as percent ± SEM of crystal violet-stained area compared to control. *P value < 0.05, ** P value < 0.001. [file 12935_2017_454_MOESM4_ESM.tif]

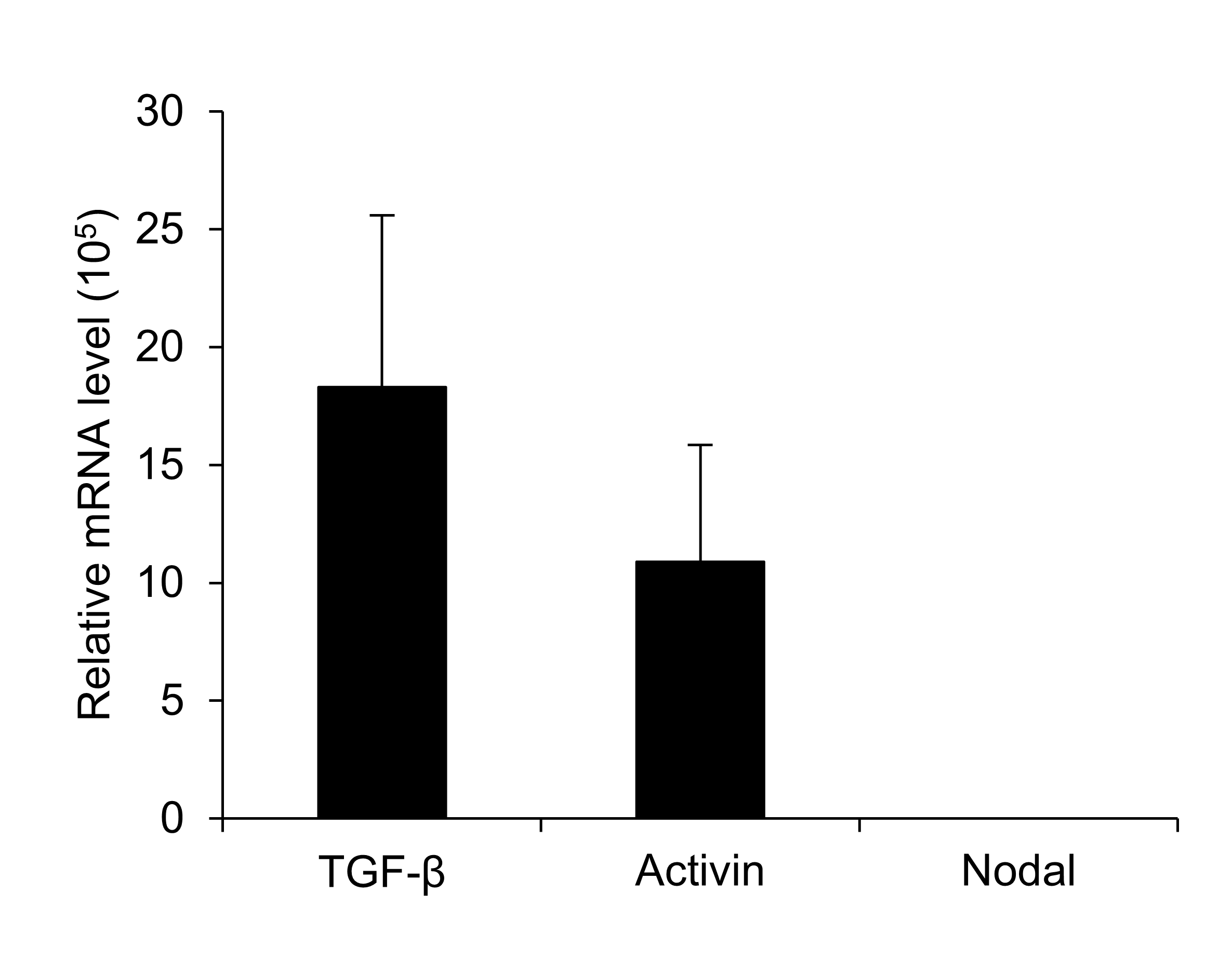

Supplement: Supplementary file 5 — Additional file 5. Steady state levels of TGF-β1, activin and nodal expression in KKU-M213 cells. Levels of TGF-β1, activin and nodal mRNA were determined by SYBR-green-based qRT-PCR using RNA extracted from 80% confluent cells cultured in 10% FBS media. Relative mRNA levels were calculated using 2−ΔCt formula compared to that of 18 s rRNA. [file 12935_2017_454_MOESM5_ESM.tif]
